# Abortion care in Haiti: A secondary analysis of demographic and health data

**DOI:** 10.1371/journal.pone.0206967

**Published:** 2018-11-08

**Authors:** Kate Meffen, Gillian Burkhardt, Susan Bartels

**Affiliations:** 1 Faculty of Arts and Sciences, Queen’s University, Kingston, Ontario, Canada; 2 Department of Obstetrics and Gynecology, University of New Mexico, Albuquerque, New Mexico, United States of America; 3 Departments of Emergency Medicine and Public Health Sciences, Queen’s University, Kingston, Ontario, Canada; Karolinska Institutet, SWEDEN

## Abstract

**Background:**

Abortion-related mortality accounts for 8% of all global maternal deaths and 97% of the estimated 25 million unsafe abortions performed each year occur in low- and middle-income countries. Haiti has the highest rate of maternal mortality in the western hemisphere and to further understand the circumstances of induced abortion in Haiti, the current work uses data from the 2012 Demographic and Health Survey (DHS) to describe the methods of induced abortion in Haiti between 2007–2012 and to identify potential factors associated with use of different abortion methods.

**Methods:**

This is a secondary analysis of nationally representative cross-sectional data from the 2012 Haitian DHS, a two-stage cluster randomized household survey. Analysis included descriptive statistics on participant demographics, methods of abortion, and location of / assistant for the abortion. Multivariate regression was conducted to determine if demographic characteristics were associated with: 1) increased or decreased odds of having an abortion; or 2) increased or decreased odds of reporting an evidence based or non-evidence based method of abortion.

**Results:**

Among the 14,287 women of childbearing age who completed the 2012 Haiti DHS survey, 289 women reported having an induced abortion in the previous five years. Recommended methods, manual vacuum aspiration (MVA) or misoprostol alone, were used in 26.6% of the abortions (n = 77). Additionally, 13.8% (n = 40) of abortions used these recommended methods in combination with a non-evidenced based method such as injections, plants or tablets. A total of 92 women had a dilation and curettage (D&C) abortion, either alone (n = 77) or in combination with another method (n = 15) and over a quarter (n = 80) of reported abortions were conducted by non-evidence based methods (n = 80). A majority of abortions using a recommended method were assisted by a relative/friend (n = 28) or were unassisted (n = 34). Most abortions occurred in private homes (n = 174) with hospitals/clinics being the second most common location (n = 84). Women in the middle (OR = 3.3, 95% CI = 2.0–5.6) and highest (OR = 7.4, 95% CI = 4.4–12.3) wealth brackets were more likely to have had an abortion in comparison to women in the lowest wealth bracket. Women who had ever been in a marital union were more likely to have had an abortion than those who had not. The only demographic factor predictive of aborting using a recommended method was living in an urban area, with urban-dwelling women being less likely to use a recommended abortion method (OR = 0.4, 95% CI = 0.2–0.9) in comparison with women living in rural settings.

**Conclusion:**

In a nationally representative survey in Haiti, 2% of women of childbearing age reported having an abortion in the five years prior to the survey. A large proportion of these abortions were carried out using non-evidence based methods and over half occurred outside of the formal health care system. Understanding women’s attitudes, knowledge and barriers around abortion is paramount to improving knowledge and access to evidence-based abortion care in an effort to decrease maternal morbidity and mortality in Haiti.

## Introduction

Abortion-related mortality accounts for an estimated 8% of all global maternal deaths [1], with an estimated 25 million unsafe abortions being performed each year, 97% of which occur in low- and middle-income countries [2]. In addition to abortion-related deaths, another 5 million women are thought to develop a disability as a result of unsafe abortion [3]. The World Health Organization (WHO) considers an abortion to be unsafe if the provider has inadequate skills to provide abortion services or the services are provided in an environment that does not conform to minimal medical standards, or both [4,5]. In many resource-limited settings women use a “concoction”, or a mixture of unsafe substances including soap, turpentine, bleach, or medications with unproven efficacy (such as quinine or oral contraceptives) to induce abortion [6]. The evidence-based abortion methods currently recommended by WHO are: a) medical abortion using mifepristone followed by misoprostol or misoprostol alone if mifepristone is unavailable, or b) a surgical abortion using manual vacuum aspiration (MVA) [3]. In 2012, WHO recommended that dilation and curettage (D&C) be replaced with MVA since MVA is associated with lower complication rates and is less painful to women [3].

More restrictive abortion laws are associated with higher levels of unsafe abortion [4]. Conversely, less restrictive abortion laws are associated with lower rates of induced abortion and very low rates of unsafe abortion [4]. Haiti is one example of a country with highly restrictive abortions laws, where abortion is legally permissible only if required to save the mother’s life [7,8]. Haiti also has the highest rate of maternal mortality in the western hemisphere with 359 deaths per 100,000 live births [9] and is one of the poorest nations in the world (ranking 163 out of 188 countries on the United Nations Human Development Index) [10]. Furthermore, the 2010 earthquake in Haiti reduced access to contraception and increased the number of unplanned pregnancies [11] with the 2012 Haitian Demographic and Health Survey (DHS) [1], reporting that 35% of married women in Haiti had an unmet need for contraception [12].

Despite the Haitian context of restricted abortion laws, high maternal mortality, and unmet need for contraception, there is little published data on abortion rates and methods in Haiti. However, in a recent quantitative analysis of abortion across 28 low and middle-income countries, Chae, et al. reported that abortion rates in Haiti were higher among married women, wealthier women, women aged 20 to 29, and women living in urban areas [13]. Qualitative data on abortion in urban areas of Haiti suggested that misoprostol and herbs were widely recognized as abortifacients and women tended to use multiple agents in combination with misoprostol to terminate pregnancies [14]. Additionally, it was reported that stigma was a significant barrier to abortion care in Haiti [14].

This secondary analysis of DHS data was undertaken to: 1) describe the methods of and circumstances surrounding induced abortions in Haiti between 2007 and 2012; and 2) identify determinants of induced abortions to better understand who is at risk for use of non-evidence based abortion methods. In contrast to prior analyses, the current work includes all induced abortions for the five-year period prior to the 2012 Haiti DHS and also considers the method of abortion. The analysis aims to help policy makers and programmers target and improve reproductive health services for women in Haiti and may be informative for those working in similar resource-limited contexts.

## Methods

### Study design

This is a secondary analysis of the 2012 Haitian DHS. Nationally representative, cross sectional data was collected from all 10 administrative departments in Haiti from January to June 2012 by the Haitian Childhood Institute [12]. In total, 23,770 Haitians were surveyed, 14,287 of whom were women aged 15–49. The 2012 Haiti DHS final report has additional details about survey design, and data collection [12]. In summary, procedures and questionnaires for DHS surveys were reviewed and approved by the ICF International Institutional Review Board (IRB), ensuring that the survey complied with the U.S. Department of Health and Human services [12]. Additionally, Le Comité National d’Éthique d’Haïti ensured that the survey complied with the laws and norms of the nation. In the first stage of sampling, clusters were established from the Fourth General Census of Population and Housing (RGPH) in 2003 [12]. Four-hundred clusters (144 in urban and 256 in rural areas) were selected systematically with probability proportional to the population size as estimated in 2011 [12]. An additional 45 clusters were selected in camp settings [12]. Informed consent was collected from each participant prior to the interview and each participant was provided with contact information should they have questions about the survey [12]. Each interview was identified only by a series of numbers (enumeration area (EA), household, and individual) and geographic coordinates were displaced at a random distance and direction up to 2 km (5 km in rural EAs) to ensure neither the individual or household could be identified [12]. All data were fully anonymized prior to download from Measure DHS. Permission to use the data was obtained from Measure DHS and the Queen’s University Health Sciences and Affiliated Teaching Hospitals Research Ethics Board approved the research protocol (Protocol # 6019938).

Demographics for all female respondents aged 15–49 are included as well details of all abortions reported within the five-year period. Any abortion that occurred between January 2007 and the date of the interview was considered to have been within the five-year period regardless of the interview date. An induced abortion is one in which a procedure is done or a medication taken to intentionally end a pregnancy. The analysis does not include spontaneous abortions.

### Data variables

The following demographic variables were included in the DHS: wealth, education level, age, marital status, residence type, and religion. These demographics may have changed between the date of the abortion and the date of interview, but the current analysis was unable to capture these differences. The wealth index was constructed by Measure DHS by weighting each asset or dwelling characteristic and standardizing the resulting wealth score to a standard normal distribution [15]. The index was provided as a wealth score separated into quintiles (poorest, poorer, middle, richer, richest) in the DHS, which were further collapsed into poor (poorest and poorer), middle, and rich (richer and richest) for the purposes of this analysis.

In the 2012 DHS survey, women were asked which method(s) was/were used for their most recent induced abortion with responses coded as: D&C, MVA, Cytotec (brand name for misoprostol), injections, tablets, plants/concoctions, and other. In this analysis, Cytotec and MVA were classified as recommended methods according to WHO guidelines, D&C was classified separately (recognizing that D&C is evidence-based, but no longer recommended by WHO guidelines), while plants, injections, tablets, concoctions and other were all considered non-evidenced based. If more than one abortion method was used the abortion was classified as multiple methods, with specific combinations of methods described in the results.

The DHS survey asked women who assisted with their most recent abortion. Possible responses included: doctor, midwife, health worker, midwife/matron with an office, matron without an office, auxiliary worker, traditional birth attendant, ougan/mambo, relative/friend, no one, and other. For this analysis, midwives and matrons were combined into a single category referred to as “midwife/matron”, while auxiliary workers, ougan/mambo (which refers to clergy of the vodou religion in Haiti) as well as other were collapsed into a single category referred to as “other”.

DHS data included the location of the woman’s most recent abortion, with possible responses being hospital/clinic, health center, centre de santé/dispensaire, family planning clinic, private doctor, a non-governmental organization (NGO) mobile clinic, house, other, and don’t know. For our purposes, health center and centre de santé/dispensaire were combined into the single category, “health centre”, while other and don’t know, were collapsed to “other”.

### Statistical analyses

Survey-specific sample weights were applied to produce representative estimates. Analysis included descriptive statistics on participant demographics, methods of abortion, and location of / assistant for the abortion. Multivariate logistic regression was conducted to determine if demographic characteristics were associated with increased or decreased odds of having an abortion using (a) any method, (b) a recommended method, (c) an abortion by D&C, (d) a non-evidenced based method or (e) a combination of more than one method. The analysis of abortion methods was restricted to women who had an abortion in the five years prior to the 2012 DHS survey and for whom complete information on their most recent abortion was available. The model controlled for wealth, education, age, marital status, residence type, religion and ever-use of contraception. To determine which demographic variables to include in the model, the Nagelkerke R Square test was used to examine the strength of the model as each demographic variable was added. A p-value of < 0.05 was taken as statistically significant. All analyses were conducted using IBM SPSS statistical software version 24.0 (IBM Corporation, Armonk, NY).

## Results

A total of 14,287 women aged 15–49 completed the 2012 Haitian DHS survey. Among these women, 501 reported at least one abortion in their lifetime and 289 reported having had an abortion in the five years prior to the survey (Table 1). Data on the method of abortion was available only for the 289 women reporting an abortion in the previous five years and the analysis is limited to this subgroup. One additional woman reported having had an abortion in the five-year period but because details about the abortion were missing, she was not included in the analysis.

**Table 1:** Demographic characteristics for all women aged 15–49 in the 2012 Haitian Demographic and health survey and for women who had had an abortion in the 5 years prior.

Demographic Variables	Number of women surveyed (N = 14287)	Women who gave birth in the last 5 years (N = 5414)	Women who reported having an abortion in the last 5 years (N = 289)
	N (%)	N (%)	N (%)
**Wealth Index**			
Poor	5457 (38.2)	2526 (46.7)	24 (8.3)
Middle	3079 (21.56	1263 (23.3)	69 (23.8)
Rich	5751 (40.3)	1625 (30.0)	197 (67.9)
**Education Level**			
No education	2282 (16.0)	1132 (20.9)	19 (6.6)
Primary	5551 (38.9)	2404 (44.4)	106 (36.6)
Secondary or higher	6454 (45.2)	1878 (34.7)	165 (56.9)
**Age Group**			
15–19 years	3475 (24.3)	394 (7.3)	27 (9.3)
20–24 years	2797 (19.6)	1239 (22.9)	69 (23.8)
25–34 years	4102 (28.7)	2407 (44.5)	128 (44.1)
35–49 years	3913 (27.4)	1374 (25.4)	66 (22.8)
**Marital Status**			
Never in union	5246 (36.7)	322 (6.0)	45 (15.5)
Married	6295 (44.1)	3811 (70.4)	129 (44.5)
Living w/ partner	1554 (10.9)	802 (14.8)	84 (29.0)
Widowed, separated, divorced	1192 (8.3)	479 (8.9)	32 (11.0)
**Residence Type**			
Rural	5470 (38.3)	3278 (60.6)	72 (24.8)
Urban	7614 (53.3)	1604 (29.6)	169 (58.3)
Camp rural	352 (2.5)	168 (3.1)	13 (4.5)
Camp urban	851 (6.0)	364 (6.7)	36 (12.4)
**Religion**			
No religious affiliation	792 (5.5)	413 (7.6)	27 (9.3)
Religious affiliation	13495 (94.5)	5001 (92.4)	262 (90.7)
**Ever Used Contraception**			
No	8192 (57.3)	2047 (37.8)	85 (29.4)
Yes	6095 (42.7)	3367 (62.2)	205 (70.9)
**Total Sample**	14287	5414	289

As shown in Table 2, of the 289 abortions in the five years prior to the survey, 26.6% (n = 77) used the recommended methods of MVA or Cytotec alone while 13.8% (n = 40) used a recommended method in combination with a non-evidenced based method such as injections, plants or tablets. In total, 31.8% of women reported having had a D&C—either by itself (n = 77), in combination with an MVA or Cytotec (n = 6), in combination with a non-evidence based method (n = 7) or in combination with both a recommended method as well as a non-evidence based method (n = 2). Over a quarter of the women in the sample reporting using non-evidence based methods for their abortions (n = 80). There were no significant differences in gestational age at time of abortion or who decided on abortion across the various methods used.

**Table 2:** Reported methods of abortion categorized as recommended, D&C, non-evidence based, or multiple methods.

Abortion Method	Number of women who reported method (N = 289)
	N (%)
**Recommended**	**77 (26.6)**
MVA	7 (2.4)
Cytotec	70 (24.2)
**D&C**	**77 (26.6)**
D&C	77 (26.6)
**Non-Evidence Based**	**80 (27.7)**
Injections	14 (4.8)
Plants/Concoctions	18 (6.2)
Injections + Tablets	2 (0.7)
Tablets	23 (8.0)
Tablets + Plants	14 (4.8)
Tablets + Other	4 (1.4)
Other	2 (0.7)
Don’t Know	3 (1.0)
**Multiple Methods**	**55 (19.0)**
MVA + Plants	1 (0.3)
Cytotec + Injections	1 (0.3)
Cytotec + Tablets	3 (1.0)
Cytotec + Tablets + Plants	2 (0.7)
Cytotec + Tablets + Others	1 (0.3)
Cytotec + Plants	11 (3.8)
Cytotec + Plants + Other	1 (0.3)
Cytotec + Other	20 (6.9)
Cytotec + D&C + Tablets	1 (0.3)
Cytotec + D&C + Plants	1 (0.3)
D&C + Injections	2 (0.7)
D&C + Tablets	4 (1.4)
D&C + Plants	1 (0.3)
D&C + MVA	1 (0.3)
D&C + Cytotec	5 (1.7)
**Total Sample**	289

Table 3 details who assisted with the abortion and the location of the abortion according to the method used. A majority of the abortions using a recommended method were assisted by a relative/friend (n = 28) or were unassisted (n = 34). Midwives, health workers, matrons and traditional birth attendants were less often identified as having assisted with abortion care. A range of providers assisted with the non-evidenced based abortions. The most common location for any type of abortion was a private home (n = 174) with hospitals/clinics being the second most common (n = 84). However, 79.2% of the abortions using D&C occurred at a hospital or clinic. Family planning clinics were not frequently accessed for abortion care (n = 3).

**Table 3:** Assistance for and location of abortion by method used.

** **	**Total Abortions (N = 289)**	**Recommended Abortion Method (N = 77)**	**D&C Abortion Method (N = 77)**	**Non-Evidence Based Abortion Method (N = 80)**	**Multiple Methods of Abortion (N = 55)**
Variables					
	N (%)	N (%)	N (%)	N (%)	N (%)
**Assistant**					
Doctor	109 (37.7)	7 (9.1)	76 (98.7)	11 (13.8)	15 (27.3)
Midwife	6 (2.1)	0 (0.0)	1 (1.3)	5 (6.3)	0 (0.0)
Health Worker	1 (0.4)	0 (0.0)	0 (0.0)	1 (1.3)	0 (0.0)
Midwife/Matron	4 (1.4)	0 (0.0)	0 (0.0)	2 (2.5)	2 (3.6)
Traditional Birth Attendant	4 (1.4)	1 (1.3)	0 (0.0)	3 (3.8)	0 (0.0)
Relative/Friend	60 (20.8)	28 (36.4)	0 (0.0)	19 (23.8)	13 (23.6)
Other	20 (6.9)	7 (9.1)	0 (0.0)	6 (7.5)	7 (12.7)
No One	85 (29.4)	34 (44.2)	0 (0.0)	33 (41.3)	18 (32.7)
**Location**					
Hospital/Clinic	84 (29.1)	5 (6.5)	61 (79.2)	7 (8.8)	11 (20.0)
Health Center	13 (4.5)	1 (1.3)	5 (6.5)	4 (5.0)	3 (5.5)
Family Planning Clinic	3 (1.0)	1 (1.3)	1 (1.3)	1 (1.3)	0 (0.0)
Private Doctor	12 (4.2)	0 (0.0)	9 (11.7)	2 (2.5)	1 (1.8)
NGO Mobile Clinic	1 (0.4)	0 (0.0)	0 (0.0)	1 (1.3)	0 (0.0)
House	174 (60.2)	69 (89.6)	1 (1.3)	64 (80.0)	40 (72.7)
Other/Don’t Know	2 (0.7)	1 (1.3)	0 (0.0)	1 (1.3)	0 (0.0)
**Total Sample**	289	77	77	80	55

Table 4 shows results from the multivariate regression estimating the probability of having an abortion at all or of having an abortion using particular methods. Women in the middle wealth bracket were more likely overall than those in the lowest income bracket to have had an abortion (OR = 3.3, 95% CI = 2.0–5.6), as were women in the highest income bracket (OR = 7.4, 95% CI = 4.4–12.3). This was likewise true when only considering D&C abortions, where women in the middle-income bracket (OR = 12.2, 95% CI = 1.1–131.7) and high-income bracket (OR = 18.0, 95% CI = 1.7–185.6) were more likely to report having had an abortion than women in the lowest income bracket. Women in the highest income bracket were more likely to use an evidence-based method of abortion in comparison to those in lower income brackets (OR = 3.7, 95% CI = 1.2 to 11.3). Women reporting lifetime use of contraceptives were also more likely to report having had an abortion (OR = 2.3, 95% CI = 1.8–2.9) in comparison to women who had never used contraception. The only demographic factor predictive of aborting using a recommended method was living in an urban area, with urban-dwelling women being less likely to use a recommended abortion method (OR = 0.4, 95% CI = 0.2–0.9) in comparison to rural-dwelling method. Compared to women who had never been in marital union, women living with a partner were more likely to have had an abortion (OR = 4.2, 95% CI = 2.9–6.1), as were women who were married (OR = 2.1, 95% CI = 1.4–3.1) and women who were widowed (OR = 3.1,95% CI 1.9–4.9). There were no significant differences in reported complications across the various abortion methods.

**Table 4:** Multivariate regression analysis estimating the probability of having an abortion at all or of having an abortion using particular methods.

Variables	Any method of abortion	Recommended method of abortion	D&C method of abortion	Non -Evidence Based method of abortion	Multiple Methods of abortion
	OR (95% C.I.)	OR (95% C.I.)	OR (95% C.I.)	OR (95% C.I.)	OR (95% C.I.)
**Wealth Index**					
Poor (ref)	1	1	1	1	1
Middle	3.3* (2.0–5.6)	0.4 (0.1–1.4)	12.2* (1.1–131.7)	1.4 (0.3–5.7)	0.3 (0.1–1.2)
Rich	7.4* (4.4–12.3)	0.7 (0.2–2.3)	18.0* (1.7–185.6)	0.5 (0.1–2.0)	0.3 (0.1–1.2)
**Education Level**					
No education (ref)	1	1	1	1	1
Primary	1.4 (0.9–2.3)	0.5 (0.2–1.4)	2.0 (0.5–8.2)	0.7 (0.2–2.3)	1.8 (0.5–6.7)
Secondary or higher	1.3 (0.8–2.1)	0.3 (0.1–0.8)	5.8 (1.5–21.7)	0.9 (0.3–2.7)	0.8 (0.2–3.1)
**Age Group**					
15–19 years (ref)	1	1	1	1	1
20–24 years	1.4 (0.9–2.2)	3.1 (1.0–10.1)	0.3* (0.1–0.9)	0.9 (0.3–2.4)	1.6 (0.4–6.4)
25–34 years	1.6* (1.0–2.4)	1.4 (0.5–4.5)	0.6 (0.3–1.6)	0.7 (0.3–1.8)	2.4 (0.6–8.8)
35–49 years	0.9 (0.6–1.4)	1.7 (0.5–6.0)	0.8 (0.3–2.3)	1.2 (0.4–3.4)	0.6 (0.1–2.7)
**Marital Status**					
Never in union (ref)	1	1	1	1	1
Married	2.1* (1.4–3.1)	2.0 (0.8–5.1)	0.8 (0.4–1.8)	0.4* (0.2–0.8)	2.4 (0.8–7.0)
Living w/ partner	4.2* (2.9–6.1)	1.6 (0.6–4.1)	0.9 (0.4–2.0)	0.6 (0.3–1.2)	2.0 (0.6–5.9)
Widowed, divorced, separated	3.1* (1.9–4.9)	0.9 (0.3–3.0)	1.1 (0.4–2.8)	0.4 (0.1–1.0)	4.1* (1.3–13.4)
**Residence Type**					
Rural (ref)	1	1	1	1	1
Urban	1.1 (0.8–1.4)	0.4* (0.2–0.9)	0.4* (0.2–0.7)	4.4* (1.9–9.9)	2.8* (1.0–7.5)
Camp rural	1.4 (0.5–4.1)	1.1 (0.1–9.6)	0.1 (0.0–8.0)	1.6 (0.1–19.7)	4.3 (0.4–49.1)
Camp urban	1.6 (0.9–2.9)	0.7 (0.2–2.5)	0.3 (0.1–1.5)	0.9 (0.2–3.9)	7.1 (1.8–27.1)
**Religion**					
No religious affiliation (ref)	1	1	1	1	1
Religious affiliation	0.8 (0.5–1.2)	1.8 (0.7–4.7)	0.9 (0.4–2.4)	0.9 (0.3–2.2)	0.7 (0.3–1.8)
**Ever Used Contraceptive**					
No (ref)	1	1	1	1	1
Yes	2.3* (1.8–2.9)	1.5 (0.8–2.9)	1.0 (0.6–1.9)	0.8 (0.5–1.5)	0.8 (0.4–1.5)

* indicates statistically significant with p-value of < 0.05.

## Discussion

Of the 14,287 women aged 15–49 who completed the 2010 DHS in Haiti, 289 or 2% reported having had an abortion in the five years prior to the interview. It is notable that over a quarter of these women used non-evidence based methods to terminate their pregnancies (27.7%) and approximately a quarter had a D&C abortion (27.7%). Guidelines recommending that D&C be replaced by MVA were published in 2012 and therefore would not have been in place at the time women in this sample were accessing abortion care (between 2007 and 2012) [3]. At the time of the DHS survey, D&C was considered a recommended method for abortion; however, we chose to classify this method separately in our analysis, to better help policy makers and programmers understand the potential need for retraining on MVA in light of the more recent guidelines. Given the time frame, this analysis does not provide any insight on the extent to which the 2012 WHO recommendation to use MVA instead of D&C for surgical abortions are being taken up by sexual and reproductive health care providers Haiti and should be an area of future study.

It was common for women in this sample to take misoprostol in addition to using non-evidenced based methods such as plants, injections or other tablets and we hypothesize that many of these women first used a non-evidenced based abortion method, and then went on to use a recommended, evidence-based method after the first attempt was unsuccessful. It is impossible to know from the DHS data which medications were included in abortifacient tablets and some women may have taken misoprostol but not known the name of the drug or recognized its trade name, Cytotec, at the time of the interview, thus introducing misclassification bias. In Haiti, misoprostol can be purchased at pharmacies without a prescription, but mifepristone is not available, in particular during the time frame of the DHS survey. Women in Haiti often gather information on abortions from community sources, and thus it is possible that specific combinations of abortifacient drugs and plants differed between geographic regions [14]. The exact content of abortifacient injections is also unclear from the DHS data although quinine and vaccines have been reportedly used to induce abortions in other contexts [6,14]. Methotrexate, an older drug used for abortions and administered as an injection, is not widely available in Haiti and is therefore unlikely to have been used to induce abortion. It is plausible that a small proportion of the injections given simultaneously with Cytotec or a D&C were prophylactic antibiotics, which was considered standard of practice at the time of the DHS survey. However, given the lack of detail in the DHS survey regarding the types of injections used, we have elected to categorize injections as non-evidenced based, particularly in light of women receiving injections alone, in which case it is possible that an ineffective or unnecessary injection was given under the pretense of terminating the pregnancy.

Results of this analysis are consistent with other published data on abortion in low- and middle-income countries. For instance, it was relatively common for women in this dataset to use more than one method of abortion (19.0%) and similar findings were described by Berry-Bibee who reported various regiments of abortifacients being used in Haiti such as chloroquine, antibiotics and other medications [14]. Additionally, women who were in marital union, women living in urban settings and wealthier women were more likely to have had an abortion. Chae, et al. similarly reported that abortion rates in Haiti were higher among married women, wealthier women, and women living in urban areas [13]. Our data expanded on Chae’s findings indicating that women living in urban areas are less likely to use evidence based methods. This analysis adds an additional insight that wealthier women were more likely to have an abortion using an evidence based method, implying that ability to pay may correlate with access to and information about services. Finally, women reporting lifetime use of contraceptives were also more likely to report having had an abortion, which may reflect higher levels of awareness about sexual and reproductive health and better access to sexual and reproductive health care among this group of women.

This analysis sheds further light on where women accessed abortion services in Haiti. Nearly all women who obtained a D&C sought care from a doctor, which would be expected as this is considered a minor surgical procedure. However, as service providers and policy makers work to shift from D&C to MVA, additional advocacy may be needed to promote task-shifting of MVA among different levels of health care professionals as advocated by the WHO [16]. Overall, the majority of women in this analysis aborted their pregnancies without accessing the formal health care system (n = 167 or 57%). Similar findings in Haiti have been published by Berry-Bibee who reported that women in Cap Hatien wishing to abort a pregnancy would first attempt a herbal remedy with or without misoprostol and if that failed, would seek assistance from a traditional birth attendant or informal health care worker. Only if the second attempt failed did women typically seek care at a formal clinic or hospital [14]. Comparable results have also been reported from eastern Democratic Republic of Congo where only 18% of women sought abortion care from a physician, nurse or midwife [17]. These results may speak to the growing body of literature that many women may choose to access oral abortifacients from pharmacies [18] and to terminate pregnancies with little health care intervention [19]. However, the DHS data did not specifically report on pharmacies and this is an area that should be considered for future study. Research gaps have been identified among women who “self-induce” abortions and more data is urgently needed on women’s preferences and experiences, access to drugs and information, and the clinical outcomes and safety for women who self-induce abortions outside the formal health care system [20].

In some settings, stigma around abortion is significant and it, along with restrictive abortion laws, can prohibit women from seeking abortion care within the formal health care setting, which in turn may lead to women seeking unsafe abortions. Berry-Bibee described high levels of religious and health-care related stigma around abortion in Haiti [14]. While the DHS survey did not provide specific data on experiences of religious or health-care-related stigma around abortion, in the current analysis, there was no association between religious affiliation and likelihood of having aborted a pregnancy.

An expert working group recently proposed a more nuanced classification of abortion safety according to the following five categories, very unsafe, unsafe, unsafe with low medical risk, safe with non-medical risk or safe [21]. This classification takes into account whether abortion is legal in the given setting, the social context and stigma associated with abortion, the qualifications of the person performing the abortion, methods used, and the degree of medical complications experienced by the woman. Given the paucity of DHS data regarding stigma, training of providers, details on the methods, and complications, it was not possible to apply a similar framework in the current analysis. We believe applying a broader, more comprehensive classification of abortion is warranted in order to better understand the associated legal and socioeconomic consequences in various settings. This would require however, that population-based data such as the DHS, be adapted to refine and substantiate the classification of abortion. Furthermore, additional research is needed to address issues of safety, as well as women’s preferences, related to self-induced abortions in both restricted and unrestricted settings [20].

This study has a number of limitations. First, there is concern about recall bias given that the abortion may have taken place up to five years prior to the interview. Additionally, demographic variables such as marital status could have changed between the time of the abortion and the day of the interview. Third, given that abortion is a highly sensitive topic and that abortion is highly stigmatized and restricted in Haiti, reporting bias may have been an issue and women may have been hesitant to report having aborted a pregnancy. Therefore, it is likely that the current figures underestimate the true prevalence of abortion in Haiti. However, others have noted that underreporting does not appear to vary by subgroups [22]. Nevertheless, as Chae et. al. reported, it is highly possible that unmarried women in particular were underreporting, thus explaining the finding that unmarried women were less likely to have reported an abortion. Fourth, the DHS survey did not collect detailed information on post-abortion complications, so the current analysis does not consider that important aspect of abortion / post-abortion care. Further research on the complications arising from abortion and experiences of stigma around abortion is needed; using mixed methods may be more useful for improving our understanding of these complex experiences. Lastly, the DHS did not collect data on the sequence in which various abortion methods were used. Although we assume that non-evidence based methods were tried first and then evidence based methods used if first attempts were unsuccessful, this cannot be determined from the current dataset. Future surveys could provide further insights on this by asking more details about the sequence of abortion methods used.

The study also has a number of strengths. The DHS collected data from all 10 administrative departments in Haiti and, therefore, included a large, nationally representative sample thereby reducing the possibility of selection bias. This study is also among the first to identify the prevalence of abortion in Haiti in a recent five-year time period and provides detailed data about the methods of abortion. Such information may help to identify interventions to address knowledge barriers and to improve access to safe, evidence-based methods of abortion for women in Haiti.

## Conclusion

In this study, 2% of women of childbearing age in Haiti who completed the 2012 DHS reported having had an abortion in the five years prior to the survey. A large proportion of these abortions were carried out using D&C, a now non-recommended abortion method according to WHO, as well as non-evidence based methods, and over half of reported abortions occurred outside of the formal health care system. Further research is needed to determine if the 2012 updated WHO guidelines have been adapted in Haiti and if MVA has become the preferred surgical abortion method. Understanding women’s attitudes, knowledge and potential barriers around induced abortion is paramount and may ultimately help to improve information on and access to evidence-based abortion methods, thereby decreasing maternal morbidity and mortality from unsafe abortion.

## Abbreviations

D&C: Dilation and curettage
CI: Confidence interval
DHS: Demographic and Health Survey
MVA: Manual vacuum aspiration
OR: Odds ratio
WHO: World Health Organizations

## References

[1] Say L, Chou D, Gemmill A, Tuncalp O, Moller AB, Daniels J, et al Global causes of maternal death: A WHO systematic analysis. Lancet Glob Heal. 2014;2: 323–333. 10.1016/S2214-109X(14)70227-X 25103301

[2] Ganatra B, Gerdts C, Rossier C, Johnson BRJ, Tuncalp O, Assifi A, et al Global, regional, and subregional classifcation of abortions by safety, 2010–14: estimates from a Bayesian hierarchical model. Lancet. 2017; 10.1016/S0140-6736(17)31794-4 PMC5711001 28964589

[3] World Health Organization. Safe abortion: technical and policy guidance for health systems, 2nd Edition Reprod Health Matters 2012; 1–123. 10.1016/S0968-8080(12)39623-7 23700650

[4] World Health Organization. Unsafe abortion: global and regional estimates of the incidence of unsafe abortion and associated mortality in 2008, 6th edition. Geneva; 2011.

[5] World Health Organization. Safe abortion: safe technical and policy guidance for health systems [Internet]. 2012 [cited 9 Mar 2017]. Available: http://apps.who.int/iris/bitstream/10665/70914/1/9789241548434_eng.pdf?ua=1 23700650

[6] Grimes DA, Benson J, Singh S, Romero M, Ganatra B, Okonofua FE, et al Unsafe abortion: the preventable pandemic. Lancet. 2006;368: 1908–1919. 10.1016/S0140-6736(06)69481-6 17126724

[7] United Nations Population Division. Abortion policy: a global review. 2002.

[8] Rights C for R. The World’s Abortion Laws 2017 [Internet]. 2017.

[9] World Health Organization. Trends in maternal mortality: 1990 to 2015—Estimates by WHO, UNICEF, UNFPA, World Bank and the United Nations Populations Division Geneva; 2015.

[10] United Nations Development Programme. Human Development Report 2015: Work for Human Development New York, NY; 2015.

[11] Behrman JA, Weitzman A. Effects of the 2010 Haiti earthquake on women’s reproductive health. Fam Plann. 2016;47: 3–17. 10.1111/j.1728-4465.2016.00045.x 27027990

[12] Ministry of Public Health and Population [le Ministère de la Santé Publique and de la Population](MSPP) HCI [l’Institut H de l’Enfance] (IHE) and II. Haïti: 2012 Mortality, Morbidity, and Service Utilization Survey Key Findings. Calverton, Maryland, USA; 2013.

[13] Chae S, Desai S, Crowell M, Sedgh G, Singh S. Characteristics of women obtaining induced abortions in selected low- and middle-income countries. PLoS One. 2017;12 10.1371/journal.pone.0172976 28355285 PMC5371299

[14] Berry-Bibee E. Assessment of induced abortion practices in urban Haiti Emory University 2014.

[15] Rutstein SO, Johnson K. The DHS Wealth Index. DHS Comparative Reports No. 6. Calverton, Maryland; 2004.

[16] World Health Organization. Health worker roles in providing safe abortion care and post-abortino contraception. 2015; 81. 26401543

[17] Rouhani SA, Scott J, Burkhardt G, Onyango MA, Haider S, Greiner A, et al A quantitative assessment of termination of sexual violence-related pregnancies in eastern Democratic Republic of Congo. Confl Health. Conflict and Health; 2016;10: 9 10.1186/s13031-016-0073-x 27053946 PMC4822240

[18] Tamang A, Puri M, Masud S, Karki DK, Khadka D, Singh M, et al Medical abortion can be provided safely and effectively by pharmacy workers trained within a harm reduction framework: Nepal Contraception 2017; 10.1016/j.contraception.2017.09.004 28935219

[19] Aiken ARA, Digol I, Trussell J, Gomperts R. Self reported outcomes and adverse events after medical abortion through online telemedicine: population based study in the Republic of Ireland and Northern Ireland. bmj BMJ BMJ. 2017;357: 2011–2011. 10.1136/bmj.j2011 28512085 PMC5431774

[20] Kapp N, Blanchard K, Coast E, Ganatra B, Harries J, Footman K, et al Developing a forward-looking agenda and methodologies for research of self- use of medical abortion. Contraception. 2017; 10.1016/j.contraception.2017.09.007 28935218

[21] Sedgh G, Filippi V, Owolabi OO, Singh SD, Askew I, Bankole A, et al Insights from an expert group meeting on the definition and measurement of unsafe abortion. Int J Gynecol Obstet. 2016;134: 104–106. 10.1016/j.ijgo.2015.11.017 27062249 PMC6434901

[22] Sundaram A, Juarez F, Bankole A, Singh S. Factors associated with abortion-seeking and obtaining a safe abortion in Ghana. Stud Fam Plann. 2012;43: 273–286. 10.1111/j.1728-4465.2012.00326.x 23239247

